# A review framework of how earthquakes trigger volcanic eruptions

**DOI:** 10.1038/s41467-021-21166-8

**Published:** 2021-02-12

**Authors:** Gilles Seropian, Ben M. Kennedy, Thomas R. Walter, Mie Ichihara, Arthur D. Jolly

**Affiliations:** 1 School of Earth and Environment, University of Canterbury, Christchurch, New Zealand; 2 GFZ German Research Center for Geosciences, Potsdam, Germany; 3 Earthquake Research Institute, University of Tokyo, Bunkyo-ku, Tokyo Japan; 4 GNS Science, Lower Hutt, New Zealand

**Keywords:** Seismology, Volcanology

## Abstract

It is generally accepted that tectonic earthquakes may trigger volcanic activity, although the underlying mechanisms are poorly constrained. Here, we review current knowledge, and introduce a novel framework to help characterize earthquake-triggering processes. This framework outlines three parameters observable at volcanoes, namely magma viscosity, open- or closed-system degassing and the presence or absence of an active hydrothermal system. Our classification illustrates that most types of volcanoes may be seismically-triggered, though require different combinations of volcanic and seismic conditions, and triggering is unlikely unless the system is primed for eruption. Seismically-triggered unrest is more common, and particularly associated with hydrothermal systems.

## Abstract (web summary)

This review dives deep into how earthquakes affect volcanoes, specifically into the relation between tectonic seismic activity and subsequent eruptions. Activity may increase in any volcanic setting in the 2–5 years following an earthquake, and especially at volcanic centres featuring vigorous hydrothermal activity.

## Introduction

Volcanic eruptions and earthquakes are amongst the most spectacular and sometimes deadliest natural events occurring on our planet, fascinating humans for centuries, with records extending to ancient times^1–3^. One naturally arising question is whether tectonic earthquakes can trigger volcanic eruptions, referred to as earthquake–volcano interactions^4^ or seismically triggered eruptions^5,6^. The statistical record of seismically triggered eruptions shows it is a relatively rare occurrence^7,8^, but understanding the causal relationships between earthquakes and volcanoes is essential towards more efficient hazard management approaches. A number of articles have summarized recent concepts and observations of earthquake–volcano interactions, including Hill et al.^4^, Koyama^9^, Manga and Brodsky^6^, Eggert and Walter^10^ and Watt et al.^11^. We herein provide a framework for using their findings and highlight recent advances. Our motivation is to identify which types of volcanoes are more susceptible to seismic triggers.

Volcanoes display an immense diversity in subsurface and aerial structure, style of eruption, chemical composition or precursory signals. As a result, the underlying seismic-triggering mechanisms may vary from one seismically triggered eruption to another. Latter^12^ already noted in 1971 that “the process is neither universal nor invariable”. Besides, earthquakes may even inhibit volcanic activity in some conditions^9,13–15^. Hence, can we identify and classify volcanoes based on their sensitivity to seismic-triggering mechanisms? A common method is to devise a classification of volcanoes based on historical records: if a given type of volcano erupts more frequently after earthquakes, then it is considered more sensitive^8,9,16,17^. Unfortunately, the limited number of recorded events precludes statistically significant correlations for most volcanoes^8^.

In this contribution, we adopt a new strategy where we start from the underlying physical mechanisms in order to derive our classification. By considering the favourable conditions for each mechanism, we construct a series of different volcano types, with each one being sensitive to different mechanisms. We then examine different earthquake scenarios and the control they exert on triggering dynamics. We thus produce a novel classification of volcanoes, according to how they can be seismically triggered aimed at informing future monitoring or statistical efforts.

## Background

### Observations

The oldest and commonest evidence of earthquake–volcano interactions is serendipitous observation^18–21^. These potentially coincidental observations later evolved into accurate records combining multiple geophysical signals^10^, permitting more precise correlations^22–25^. In particular, the recent emergence of satellite monitoring as a reliable tool in geosciences allowed for a more systematic and consistent monitoring of volcanoes globally^17,26–28^. The influence of an earthquake on an eruption can also be inferred a posteriori from crystal textures^29–31^. Like other authors, we emphasize that a spatio-temporal correlation between seismic and volcanic events does not necessarily imply a causal relationship. Two concurrent events could result from a common third underlying process, or occur by chance. Yet observing a correlation is a necessary first step in unravelling a potential causal relationship.

There are documented cases of both changes from quiescence to eruption^6,20,29,32–35^, and changes in style of an ongoing eruption^36–40^ in the weeks following an earthquake. Earthquakes may also trigger a broad spectrum of non-eruptive unrest phenomena including increased seismicity^25,41–45^, degassing^17,25,34,46,47^, heat flux^26–28,48^ or subsidence^49,50^. Hydrothermal systems are particularly sensitive to seismic stimuli, with many reports of increased activity following earthquakes^6,51–55^.

### Statistical inference

Statistical tests are a crucial step to assess whether eruptions follow earthquakes due to significant coupling or simply by coincidence. Determining the temporal and spatial extent of earthquakes’ impact on volcanoes also helps to constrain plausible mechanisms and inform hazard models. The historical earthquake and eruption records are regarded to be complete only in the most recent period (1960s–present)^8,56^. As a result, the sample size (i.e. number of events considered) is quite small, decreasing the robustness of statistical tests.

The early statistical groundwork on a global scale^5,6,10,56,57^ was updated by Sawi and Manga^8^ who conclude that the apparent correlations within a few days occur most likely due to chance. Yet, there exists a slight but significant increase in eruption rate in the 2–5 years period following an earthquake on a global scale^8,33,56^. Statistical tests perform better on regional scales, in particular in active subduction zones^8,10^. This is supported by detailed studies of the Chilean^11,16^, Japanese^58^ and Indonesian^7,33^ subduction zones which all show increased eruption rates—to various degrees—following large magnitude earthquakes. Ultimately, the internal state of the volcano—whether it is “ready” to erupt—dictates whether it will be seismically triggered or not^7^. This association may thus simply reflect the fact that subduction zones feature the highest concentration of large earthquakes and volcanoes on the verge of erupting^7^.

The maximum distance at which eruptions may be seismically triggered is debated. Delle Donne et al.^27^ propose a distance–magnitude relationship, akin to what is found in the mud volcanism and soil liquefaction literature^59,60^. For triggered-eruptions stricto sensu, Nishimura^56^ suggests a limit of about 200 km from the epicentre, whereas Marzocchi et al.^61^ propose 1000 km. Concerning triggered-unrest, earthquake-induced thermal anomalies^27,28^ and seismicity^62^ are both reported at distances over 10,000 km. This matches observations of seismically induced changes in groundwater level or streamflow^63^.

## Triggering mechanisms

**Fig. 1:**
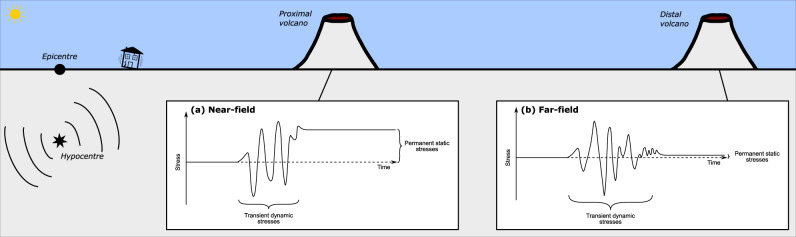
The difference between dynamic and static stresses. Dynamic stresses are transient, whereas static stresses last permanently. **a** In the region close to the epicentre, dynamic and static stresses have similar amplitudes. **b** Far from the epicentre, however, dynamic stresses have a much larger amplitude and static stresses are negligible.

A wide range of triggering mechanisms have been proposed, affecting the host rock, magma chamber and/or hydrothermal system, causing stress changes in solid state and dynamic variations in fluid and multiphase systems^4,6,9,64^. Earthquakes result from sudden ruptures in the crust, and cause significant changes in the surrounding stress field. We must thus investigate how stress perturbations affect magmatic systems in order to understand how this may eventually lead to an eruption. Stress can be transferred either statically or dynamically (Fig. 1), which will in turn dictate what triggering mechanisms can occur. We now examine both cases.

### Static processes

Static stress changes result from the deformation of rocks following an earthquake^65^ (Fig. 1). The resultant strain is spatially confined to within a few fault lengths around the epicentre and remains until the stress is elastically released or dissipated by ductile flow. Static stress changes can be either compressive or extensional, both of which might promote eruption^9,56^.

**Fig. 2:**
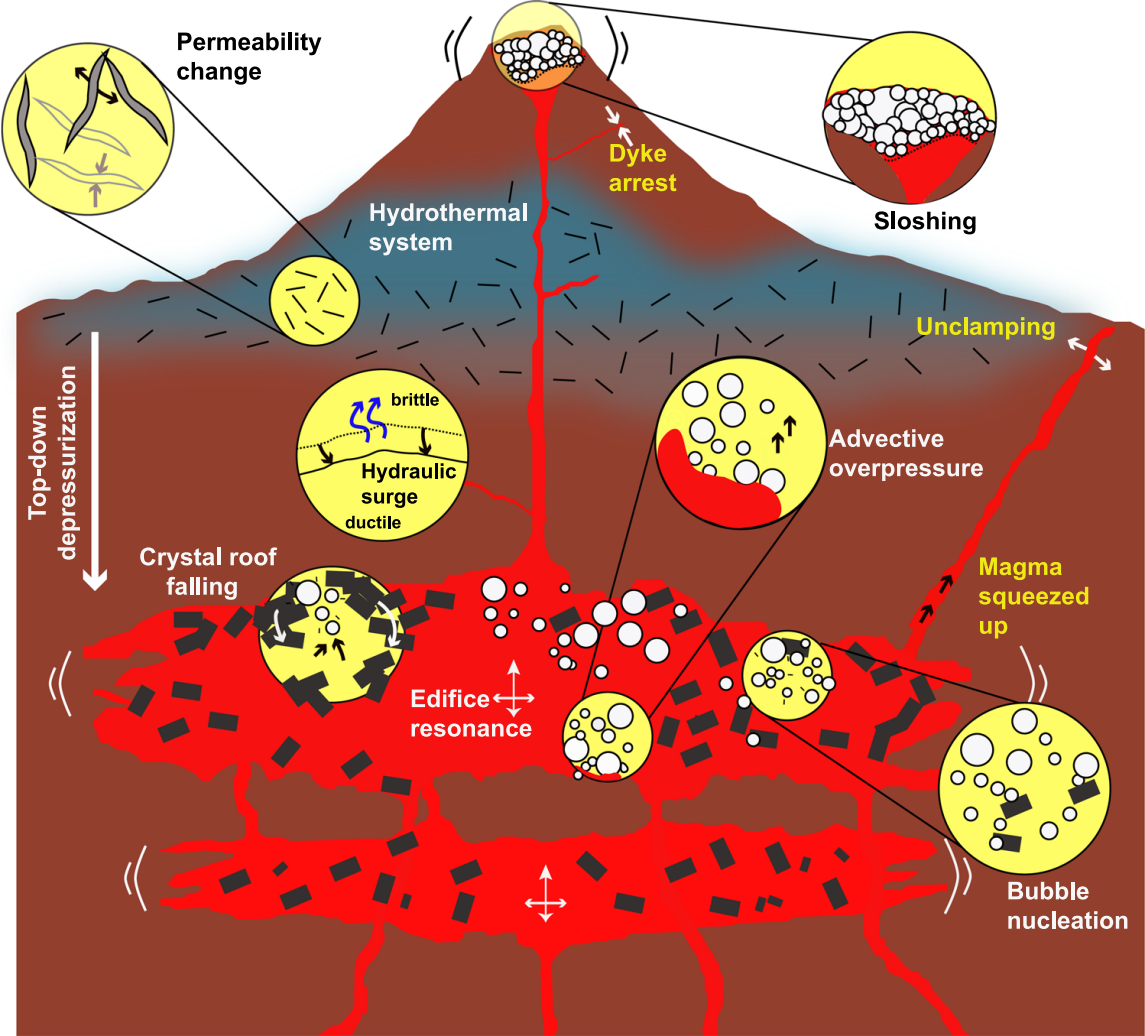
The most common seismic-triggering mechanisms. Mechanisms names in yellow are due to static stresses whereas those in black and white arise from dynamic stresses.

Extensional stresses facilitate dyke opening by “unclamping” the system (Fig. 2), thus promoting magma transport and potentially triggering an eruption^16,29,32,33,66–75^. If the magma already contains bubbles, then reduced compression could result in overpressure^76,77^. Additionally, extension favours strike-slip faulting and increased permeability^78,79^, thus allowing increased advection of magmatic fluids and melt^15,78^.

Compressional stresses have been conceptualized to squeeze magma upwards^13,66,67,80–83^ (Fig. 2), though this process has been criticized^84^. Conversely, compression at shallow depths has been inferred to inhibit and arrest dyke propagation^9,14,85–87^, and thus prevent eruption. We emphasize that the orientation of volcanic structures relative to stresses is critical to these models.

Seismic stresses are eventually dissipated by viscous flow of the lower lithosphere and asthenosphere^88,89^. This process happens over years to decades. This slow relaxation of underlying layers will impose “quasi-static” stress changes on the overlying, brittle lithosphere. These quasi-static stress changes have also been postulated to favour volcanic eruptions^4,61^, though the physical mechanism(s) require further study.

### Dynamic processes

Dynamic stresses involve the oscillatory stresses induced by seismic waves^90^ (Fig. 1). The amplitudes of dynamic stresses decrease much more gradually with distance than their static counterparts, hence dynamic stresses will generally (1) be greater and (2) travel much further than static stresses^6,90–92^. However, dynamic stresses are oscillatory phenomena, and thus feature a range of characteristic frequencies and are transient. Responses to dynamic stresses can be broadly divided into three categories.

#### Volatile processes

Volatiles have the lowest viscosities of all volcanic fluids (as low as 10^−5^ Pa.s), high compressibility, and can thus respond quickly to dynamic stresses. Three mechanisms associated with volatiles could lead to an eruption, namely (1) bubble nucleation and growth, (2) advective overpressure associated with bubble rise and (3) falling crystal roofs facilitating vesiculation (Fig. 2).

Dynamic stresses could induce bubble nucleation in magmas^6,23^ by varying the local pressure. The phenomenon is well known in other fluids, in particular in water^93^, and usually referred to as cavitation. Static decompression may sometimes be sufficient to trigger nucleation^23,33,35^. Pressure oscillations in the fluid can locally change the solubility and diffusivity of volatiles (mostly water), thus accelerating bubble formation processes. The phenomenon was experimentally demonstrated for groundwater^94^. Bubble nucleation in magma, however, is a very complex process, depending upon many parameters^95^ (e.g., volatile oversaturation, melt composition, presence of nucleation sites). For instance, it is notoriously difficult to nucleate bubbles experimentally in crystal poor rhyolite^96^, sometimes requiring immense pressure drops >100 MPa, much larger than seismic dynamic stresses^6,90^ (generally <10 MPa). With abundant nucleation sites, however, a few MPa may be sufficient to induce bubble nucleation^97,98^. To the best of our knowledge, there is no available experimental evidence showing that pressure oscillations can induce bubble nucleation in silicate melts.

Magma often already contains bubbles, in which case problems related to nucleation become irrelevant. Dynamic stresses may then accelerate volatile diffusion and bubble growth. Rectified diffusion is a very commonly cited mechanism where seismic waves enhance diffusion of volatiles inside the bubbles^99–101^ but its effects have initially been overestimated, and it is now considered most likely inefficient in magma^6,102^. We will then not consider rectified diffusion further in this contribution. Other dynamic mechanisms that could facilitate bubble growth and coalescence include Ostwald ripening^103,104^ or advection due to Bjerknes force^105^, but they have not been considered under the lens of earthquake–volcano interactions yet.

A further mechanism related to bubbles is advective overpressure^106,107^ (Fig. 2). Here, it is considered that bubbles that were previously held down to the reservoir floor or walls by surface tension can be shaken loose by seismic waves. Assuming an incompressible fluid, a closed system with undeformable walls and no mass exchange between the bubbles and the melt, the bubbles can carry large overpressures with them while rising^106,108,109^. Nevertheless, this mechanism has been heavily debated^110,111^ because most of the assumptions are unrealistic for a magmatic reservoir. Advective overpressure could still be effective in hydrothermal and hydrogeological systems however^53,54,107,112^. It is also possible that seismic waves increase bubble rise ascent speed, as shown by preliminary results from analogue experiments in shear thinning fluids^113,114^.

Finally, seismic waves could dislodge crystal aggregates that accumulate on top of a magma reservoir^4,6^ (Fig. 2). Upon detaching, dense crystals will sink and lighter melt will rise to replace them. Upwelling melt is then prone to vesiculation, which could in turn lead to an eruption. Preliminary calculations^4,6^ showed the mechanism to be theoretically realistic, but it will require further work to demonstrate that (a) these crystal roofs exist, (b) characterize their rheology and (c) validate the theory used to describe sinking of crystal plumes.

#### Resonance processes

The second category of mechanisms relates to the mechanical sway of magma in response to shaking. The amount of movement will depend on a number of parameters, including the resonant frequency of the system, mainly controlled by the edifice dimensions and reservoir geometry. If the seismic waves match this frequency, the processes will be greatly enhanced; otherwise effects will be negligible. Two resonance mechanisms have been proposed, namely sloshing and edifice resonance (Fig. 2).

Seismic waves can induce sloshing in the reservoir^115^. Sloshing refers to the movement of a fluid inside its container^116–118^—here magma in its reservoir. Sloshing of a foam layer in a magmatic reservoir or conduit could lead to foam collapse, increased degassing and vesiculation, potentially forming gas slugs and Strombolian eruptions^115^. Analogue experiments show that foam collapse will only occur if (1) the incident seismic waves carry significant energy around the reservoir’s resonance frequency to initiate sloshing and (2) a magma foam is present with a free top surface or is overlying a deeper dense melt region^115^. The mechanism will further be facilitated by a foam featuring a high bubble fraction, large bubbles and low melt viscosity.

Seismic waves can also increase melt and volatile migration inside a volcanic edifice^119^. A combination of analogue and numerical models show that shaking will accelerate fluid movement in either direction (upwards, downwards or laterally) depending upon the fluid buoyancy and storage depth^119^. Lighter (in particular bubble rich) and shallower fluids will tend to move upwards. The phenomenon is again greatly enhanced when the seismic waves resonate with the edifice. Thus, edifice resonance may either favour eruption by facilitating magma mobilization upwards or retard an eruption by forcing magma downwards^119^.

#### Hydrothermal system triggering

Hydrothermal and geothermal systems have been observed to be extremely sensitive to earthquakes and dynamic stresses in particular^6,15,41,51,52,54,90,120–132^. Hydrothermal systems are also generally well connected to the underlying magmatic reservoir^133,134^, hence destabilization may lead to a top-down depressurization of the entire magmatic system, and eventually magmatic eruption^4,15,135–138^ (Fig. 2). The possibility for hydrothermal systems to form a link between seismic waves and magmatic reservoir destabilization has received little attention under the lens of seismic triggers^4,83,138^ but observations^15,34,42,127^ suggest that it could play a major role. The triggering mechanisms of hydrothermal systems generally fall into two categories: change in fluid pressure and change in permeability.

The physical models describing changes in fluid pressure in hydrothermal systems are similar to the ones described in section “Volatile processes”. The main mechanisms are bubble nucleation^94^ and advective overpressure^54,107,112^. Here, however, the main fluid is water, with a viscosity of 10^−3^ Pa.s. Additionally, the gas phase is generated by evaporation instead of volatile diffusion in the liquid phase. These features allow for much faster kinetics and less viscous dissipation, making these mechanisms more efficient in hydrothermal settings.

Seismic waves can significantly alter permeability over short time-intervals^59,139^ (Fig. 2). Such changes have been particularly well observed in hydrogeologic systems^140^, with sudden variations in streamflow^141,142^, groundwater level^143–147^, temperature^148–150^ and seismically triggered mud volcanism^6,60,151–153^. With increased permeability, regions of higher and lower pressures may become connected, allowing fluid flow and pore pressure redistribution^59,63^. The sudden influx of fluids into originally low-pressure zones, may push such regions beyond a critical pressure threshold and produce an eruption^120^. On the other hand, reduced permeability allows local pressurization, which may lead to fragmentation and eruption^154,155^. The mechanisms described hereafter are particularly attractive because they necessitate relatively small (<1 MPa) dynamic stresses, and may thus be triggered more easily^59^. Three mechanisms are often invoked to explain changes in permeability.

Firstly, seismic waves may unclog or clog fractures^156–160^. The passage of seismic waves may intensify fluid flow, which could in turn entrain small particulates resulting in both clogging^160^ and unclogging^156^ downstream depending on fracture orientations.

Dynamic stresses may also enhance or reduce permeability by opening, closing or shearing cracks^15,132,141,161–163^. Opening new cracks or widening already existing ones increases permeability whereas other fractures with less favourable orientations would be closed and hence decrease permeability.

Finally, seismic waves may lower the brittle-plastic transition between the hydrothermal and magmatic systems^4,137^ (Fig. 2). For this scenario, we assume that there exists an impermeable plastic transition zone underlying the hydrothermal system retaining pressurized fluids^164^. In this case, the strain rates imposed by seismic waves may be sufficient to promote brittle behaviour and release overpressurized fluids into the hydrothermal system (referred to as a hydraulic surge^4^), favouring unrest and eruption^4,137^.

### External triggers

Earthquakes can also trigger eruptions indirectly via external triggers. These occur when an earthquake triggers a non-volcanic event which then cascades towards an eruption. A typical example is that of an earthquake triggering a landslide or block and ash flow above a critically pressured magmatic reservoir or dome^165–167^. The resulting sudden decompression may lead to eruption of magma. Another documented external trigger is via crust decarbonation^40^. Earthquakes induce cracking in the crust underlying the magmatic reservoir, thus releasing important volumes of CO_2_. CO_2_ then flushes the reservoir, significantly lowering the solubility of water, hence triggering vesiculation, pressurization and eventually producing the observed changes in eruption style. While it is important to explore the possible feedbacks between magmatic systems and their environments, we will not consider external triggers further, and solely focus on direct interactions between earthquakes and magmatic systems.

External triggers hence have the potential to trigger eruptions in many possible ways, it is thus important to explore, in the future, the possible feedbacks between an igneous system, its hydrothermal and their mechanical environment when subjected to an earthquake.

## Volcano types

For each mechanism, there is a set of favourable physical parameters maximizing triggering efficiency. They can be divided in two categories: (1) volcanic (e.g. melt viscosity) and (2) seismic (e.g. seismic wave frequency). We examine volcanic parameters first.

Our choice of parameters is developed by capturing both the important complexity associated with the mechanisms whilst remaining simple enough to be applied. From section “Triggering mechanisms”, we see that two parameters, namely magma viscosity and whether the system is open or closed, play critical roles for many mechanisms. The sensitivity of hydrothermal systems make them the key third parameter to be. It is interesting to note that this choice of parameters which naturally arises from our analysis resembles other recent volcano classifications^168,169^, despite the different objectives. Our classification currently focuses on subaerial volcanism only. Seismic events do trigger various responses from submarine volcanoes^33,170–172^; however, the current amount of available data are too scarce to be fitted in our classification.

The first parameter is magma viscosity, for which we will consider two limiting cases, namely low and high viscosity. Low viscosities generally correspond to basaltic, crystal poor magma, and are lower than or equal to ~10^4^ Pa.s^173,174^. We delineate high viscosities as greater than or equal to ~10^5^ Pa.s and can be achieved by rhyolitic melt, or through the addition of suspended crystals and bubbles^175,176^. Other parameters such as water content or temperature also exert significant control on viscosity. This simplified classification does overlook some major rheological properties (e.g. non-Newtonian behaviour^177,178^) yet captures the magma properties that favour certain mechanisms. For instance, it is very unlikely for a magma with viscosity 10^5^ Pa.s to ever experience sloshing, but one with viscosity 10^1^ Pa.s will.

The second parameter is whether the system is open or closed to degassing. Open systems are permeable and volatiles can easily escape as bubbles, directly out of magma or through fractures. In contrast, in a closed system, permeability is low and volatiles are trapped and cannot leave. It is quite rare for a volcano to display pure open- or closed-system degassing, yet end members are observed in nature for both cases. Lava lakes or Strombolian-style volcanoes are typical examples of open systems. Many very explosive eruptions show a phase of closed-system degassing with low levels of degassing prior to eruption^169^. It is very common for volcanoes to transition from open to closed through time, and even within a single eruptive phase, via a wide range of processes^154,179–183^. It is key to note that our definition of open and closed systems relates to shallow volatile-controlling conditions and differ from many petrological studies which refer to the connection between deep magma sources and shallow systems.

The third volcanic parameter considered is the presence of an active hydrothermal system. Arguably every volcano features a hydrothermal system; however, we define distinct end members of how well developed the hydrothermal system is. Some volcanoes display clear, persistent hydrothermal surface activity—e.g. acidic crater lakes, and fumaroles—whereas others show barely any visual sign of activity. For instance, it is quite common for smaller hydrothermal systems to dry due to proximity with magmatic bodies^169^. Thus, for the purpose of this classification, we will consider that a developed hydrothermal system is either present or absent.

We have chosen three parameters, each with two end-member cases, thus yielding 2^3^ = 8 possible combinations. Each combination corresponds to a type of volcano that will be susceptible to different triggering mechanisms. For the purpose of brevity, we limit the analysis to the five most common types, shown in Table 1. For each type, we provide a natural example and a list of the most efficient triggering mechanisms (Table 1). Importantly, volcanoes are not fixed in a given type but rather move between different categories with time, for example bimodal volcanoes can erupt two different viscosity magmas (e.g. Yellowstone, USA^184^). Timescales for these changes span orders of magnitudes from minutes (e.g. bubble nucleation) to millennia (e.g. fractional crystallization).

**Table 1:** The five most common volcano types according to our classification.

For each type, we propose a natural example and list the possible triggering mechanisms. The three volcanic criteria are indicated with text and a diagram: (1) bright red stands for low viscosity, dark red corresponds to high viscosity, (2) a conduit extending to the surface represents open system whereas a conduit stopping at depth represents closed system, (3) the presence of a hydrothermal system is indicated by the initials “HS” and a blue region with circular arrows above the magma chamber, the lack thereof indicates absence of a hydrothermal system.

## Earthquakes

The characteristics of the stress perturbation also play a major role in determining whether a given mechanism will trigger activity^6^. These characteristics will result from a combination of (a) the earthquake’s attributes and (b) the volcano’s location with respect to the epicentre. Each earthquake features a unique set of characteristics (e.g. magnitude, focal mechanism, depth), and its effects can be dramatically different from one location to another^185^. This will depend mainly on distance to the epicentre but also direction and local site amplification factors^186,187^. Moreover, some physical mechanisms are sensitive to the frequency of incoming seismic waves^115,119^. Hence, our choice of parameters should reflect this complexity whilst being simple enough for our classification to be effective. As for section “Volcano types”, we have chosen three keys: (1) peak ground velocity, (2) frequency and (3) static stress change amplitude. We consider two possible end-member values for each one, yielding 2^3^ = 8 different scenarios.

The first parameter is peak ground velocity (PGV), referring to the largest shaking speed effectively felt at the magma reservoir location. This value will generally depend on a number of variables such as earthquake magnitude, distance and direction to the hypocentre, or country rock structure. For example, higher magnitudes and shorter distances will generally yield stronger PGVs (and vice versa). The distribution of PGVs can be directional, with stronger PGVs in the rupture direction^147,185^. There may also be an increase in PGV at certain great distances from the epicentre due to SmS arrivals^186,188–190^. Similarly, seismic waves may be focused by local crustal heterogeneities or amplified by topographic irregularities, thus resulting in significantly higher local PGVs^42,187,191–194^. We focus on two possible cases: strong and moderate PGVs. We adopt the criterion that strong PGVs may produce a magmatic response, whereas moderate PGVs only affect hydrothermal systems^15,132^ (and implicitly assume that there may exist very weak PGVs that do not trigger any response). This choice is motivated by field observations that hydrothermal systems are triggered by much smaller dynamic stresses than magmatic systems^6,15,51,59,60,195^.

The second parameter is frequency (or alternatively wave-period). Most processes are greatly enhanced when the driving frequency approaches the system’s resonance frequency. The resonance frequency of a magmatic reservoir depends on its size, geometry and the acoustic property of fluid^196^. It is generally in the range 0.001–1 Hz^115,119,196^. On the other hand, hydrothermal systems have higher resonance frequencies—typically 0.5–5 Hz^196,197^. Most local earthquakes exhibit frequencies related to the propagation of surface wave components in the range 1–10 Hz, but only very large earthquakes display frequencies components below 1 Hz. Frequency is particularly relevant for melt and volatiles processes. For instance, sloshing and edifice resonance have been shown to occur only at very low frequencies^115,119^. Regarding bubble nucleation and growth processes, experimental results on magma are unavailable, but analogy with other low viscosity fluids suggests that frequency plays a capital role^94^. Regarding permeability changes in hydrothermal systems, frequency of incoming waves is also a crucial parameter, regardless of the mechanism considered^59,159,198^. Hence, we consider two limiting cases, low and high frequencies, with the low-frequency condition being matched when frequencies lower than 1 Hz are present. These frequency choices encompass two important frequency ranges for seismicity at volcanic systems; so called long-period (LP) earthquakes and tremor (at 2–5 Hz) and very-long period (VLP) seismicity (at 0.1–0.03 Hz) and hence relate to the periods of natural excitation and resonance in the volcanic edifice.

The third parameter is static stress change amplitude. Static stress changes are highly directional, i.e. the type and magnitude of the change will strongly depend on the azimuth with respect to the epicentre. Static stress changes at and around a volcano can be numerically computed^199^, and such approaches have been successfully applied to a multitude of volcanic complexes such as Mt. Fuji^72^, Kilauea^200^, Mauna Loa^69^, Pinatubo^83^, Karymsky^70^, Cerro Negro^29,68^, Sierra Negra^75^, Copahue^73^, Vesuvius^67^, Mt. Etna^34,201^, Merapi^202^, Sinabung^203^, Mt Aso^74^ and Stromboli^34^. We follow the generally accepted view that large static stress changes, both extensional and compressional, have the potential to trigger significant volcanic responses, whereas small stresses will be mostly negligible and leave the system unchanged. It is common to use 10 kPa as a limit under which static stresses can be considered negligible, since this is approximately the magnitude of ocean tidal stresses^6^. For our classification, we restrict our attention to two end-member cases, namely large (>10 kPa) and low (<10 kPa) amplitudes.

## Discussion

### A new framework to examine seismic triggering of volcanoes

We have identified five common volcanic types as well as eight different seismic scenarios, yielding a total of 5 × 8 = 40 possible combinations. For each of these 40 cases, we examine the viable seismic-triggering mechanisms with the given conditions. The results are presented in Table 2, which then constitutes a first-order classification of volcanoes, based on physical mechanisms with the potential for seismic-triggering.

**Table 2:** Possible eruption-triggering mechanisms for each of the five volcano types and different earthquake scenarios.

The three parameters for each earthquake scenario are represented by bar diagrams. A black bar indicates whether the parameter is high (top), moderate (middle) or low (bottom), whereas a grey area signifies that the parameter can take any value. “PGV”, “f” and “SSCA”, respectively, stand for “peak ground velocity”, “frequency” and “static stress change amplitude”. The symbols used for volcano types are identical to Table 1.

Each column in Table 2 represents one of the five different volcano types defined in section “Volcano types”, each featuring a different set of our three volcanic parameters. Each line in Table 2 represents a different earthquake scenario, based on the three seismic parameters discussed in section “Earthquakes”. Each cell from Table 2 therefore illustrates a unique combination of an earthquake scenario with a specific volcano type, and lists the relevant mechanisms. This does not indicate that these mechanisms will necessarily happen in the case of a corresponding earthquake, but is rather an indication of which mechanisms are physically realistic with the given set of parameters. By extension, absent mechanisms are unlikely to trigger eruptions.

The first key outcome from Table 2 is that there is no empty column, meaning that all the volcano types presented may be seismically triggered. This result matches previous historical observations^8,10,16,33,56^ that seismic triggering can happen for any type of volcano, in any tectonic setting. Furthermore, recent observations suggest that fluid movements dominate the response in the case of low-viscosity systems, whereas for more viscous systems, elastic processes may play a more important role^17,204^. This discrepancy is also captured in Table 2: the low-viscosity columns feature many more mechanisms associated with fluid movements (e.g., sloshing, advective overpressure, shaking-induced migration) than the high-viscosity cases.

Table 2 also reinforces the role of hydrothermal systems. Many studies highlight that hydrothermal areas are particularly sensitive to seismic perturbations^6,42,48,52,53,128,156,170^. Hydrothermal systems sit at a strategic location and constitute a key link between magmatic reservoirs and their environments. As such, it is important to carefully examine their role in eruption triggering^4,138^, and in particular, as intermediate between tectonic earthquakes and volcanic eruptions.

The second key observation from Table 2 is the disparity in the numbers of mechanisms, with values ranging between zero and six. It is tempting to directly relate the number of mechanisms in a cell to the likelihood of a seismically triggered eruption to occur, but this would most likely be erroneous. Table 2 does not include any quality assessment, i.e. it does not mention how efficient and how well understood is each mechanism. Some mechanisms are supported by an extensive range of theoretical and experimental data (e.g. static triggers, permeability changes in hydrothermal systems). Others rely on analogy or idealized assumptions (e.g. advective overpressure, bubble nucleation, falling crystal roofs). Section “Triggering mechanisms” provides some information on what supporting data are available for each mechanism and more quantitative considerations can be found in previous reviews^4,6,59^. A future improvement of this work could be to define and compute an effectiveness parameter for each mechanism. Another interesting future step would be to consider whether different mechanisms may occur simultaneously, interact and possibly compound their effects^64^.

It is a delicate task to assess whether a given eruption was seismically triggered^6,24^, but it is even more challenging to identify the responsible mechanism(s). The three volcanic parameters presented here can be assessed for most eruptions, thus constraining the possible mechanisms. These mechanisms may then be tested for validity. For instance, static stress changes can be computed numerically^35,69,72,73^, occurrence of resonant oscillations can be measured, crystal and bubble textures may retain a signature of nucleation events, and hydrothermal processes can be constrained with seismic monitoring^205^ and electromagnetic surveys^206^. Constraining the triggering mechanisms for a given type of volcano will be useful for future statistical studies, but may also inform and guide monitoring strategies and hazard assessment.

### The importance of critical state

A significant number of cells in Table 2 are empty (six, excluding the last line). This corroborates observations that seismically triggered eruptions are a rare occurrence^7^. One of the reasons why earthquakes do not trigger more volcanic eruptions, and why some very large earthquakes trigger very little activity (e.g. Sumatra M9.2 2004 earthquake^6^), is that the range of conditions where seismic triggering is possible forms a very narrow window that is not often met^7,35^. In particular, many studies have highlighted the need for a volcano to already be in a critical state in order to be seismically triggered^4,6,7,10,13,19,25,27,32,33,48,56,57,137,207^. Conceptually, a volcano may be considered in a critical state if it is close to erupting. This concept is particularly difficult to quantify, since criticality may take different forms at different volcanoes. For instance, Manga and Brodsky^6^ compare the overpressure in the magma chamber to the necessary tensile stress to initiate and sustain dyking to broadly estimate the degree of criticality. Bebbington and Marzocchi^7^ propose a “clock advance” mechanism, similar to what is found in the triggered-seismicity literature^208^. In this view, earthquakes merely accelerate the countdown to the next, inevitable eruption. Seismically triggered eruptions are then a consequence of a volcano being particularly advanced in its cycle towards eruption. For instance, the 1996 simultaneous eruptions of Karymsky Volcano and Akademia Nauk volcano occurred 2 days after an M_w_7.1 earthquake, but also marked the end of 14 years of continuous inflation^70^. This concept also offers an explanation to why the M9.2 Sumatra 2004 earthquake did not seem to trigger any eruption, despite being located in one of the most active volcanic zones in the world^6,33^. It is possible that none of the nearby volcanoes were close to erupting at the time. Although rarely reported, it appears that volcanoes that are not in a critical state can still be seismically triggered into eruption. For example, La Femina et al.^29^ describe the deposit of an eruption that would have seemingly not been possible to erupt without an earthquake. It is a formidable task to provide a universal definition of critical state, and something to consider for government agencies when assigning alert levels^209^ as it is important for seismic triggering. At the very least, the presence of magma within reasonable distance to the surface appears to be a necessary condition, without which the cases presented in Table 2 are less relevant.

The discussion above highlights the difficulty to define what constitutes a seismically triggered eruption. An eruption is the culmination of a cascade of intertwined processes (magma generation, transport, storage, pressurization, fragmentation, etc…), and a single tectonic event cannot be held responsible for this entire chain. Earthquakes may have a less direct influence, and impact many of the different steps towards eruption (e.g. magma generation or melt segregation). For instance, seismic waves may play a key role in unlocking mushes^210–213^ or promoting diapirs via instabilities^214^. These processes will in turn exert some control of the timing and style of eruption, but here we consider that such mechanisms do not trigger eruptions stricto sensu and thus lie beyond the scope of this study. We do nonetheless acknowledge their important role. Similarly, we mentioned the case of external triggers in section “External triggers”—sequences of events where an earthquake triggers a non-volcanic phenomenon which itself causes an eruption, and why we do not take them into account in our classification. Furthermore, for both the 1707 Fuji and 1991 Pinatubo eruptions, it was suggested that the static stress perturbation from an earthquake allowed basaltic magma to intrude into a dacitic reservoir, leading to magma mixing and eventually a Plinian eruption^72,83^. The static stress mechanism does fit within our classification, but it did not trigger the eruption per se. An elegant solution proposed by Marzocchi^57^ is to use the term “promote” rather than “trigger” in order to emphasize the complex nature of these processes.

### Changes in unrest vs. eruption

A key observation from natural events is that earthquakes trigger a change in unrest more often than a magmatic eruption^17,22,27,28,42,215,216^. Here, we adopt the general view that unrest refers to any deviation from baseline behaviour and can take various geophysical or geochemical forms^168,217,218^. In the special case of seismically triggered unrest, volcanic seismicity is, by far, the most commonly reported phenomenon^42,45,62,122,132,219,220^. Other reported processes may include increased degassing^17,47^, changes in fumarolic activity^45,48^, thermal activity^26–28^ or gas chemistry^46^. Reports and information about unrest are not as well reported as eruption data, due to the subjective definition of “baseline behaviour” or the lesser impact unrest may cause to surrounding communities^218^. Yet, unrest episodes are generally directly identified as being related to the earthquakes (most often on the basis of spatio-temporal coincidence with the passage of seismic waves), whereas eruptions generally receive more careful statistical and physical analyses before a correlation is established^24^. Seismically triggered unrest is thus quite commonly accepted whereas seismically triggered eruptions retain some controversy.

For our purposes, a decisive question is whether the mechanisms responsible for triggered-unrest differ from the ones discussed previously. Many reports highlight that the onset of unrest seems to match seismic waves arrival, suggesting a dynamic origin^22,47,48,52,132,202^. For instance, triggered seismicity is likely caused by small changes in permeability in hydrothermal systems, allowing geothermal fluids to migrate and change the local stress state^90^. Therefore, unrest may be triggered without the magmatic system being in a critical state, hence explaining the more frequent occurrence of triggered-unrest compared to triggered-eruptions.

### Future directions

Our framework highlights that hydrothermal systems may be more sensitive than magma to changes induced by seismic activity. This could lead to heightened unrest in the hydrothermal system and to eruption if the magmatic system is in a critical state. The link between hydrothermal systems and magmatic systems is a key area for future research, with a particular focus on the role of fracture formation, fluid migration and wave propagation through the hydrothermal and magmatic systems and in particular the magma-hydrothermal transition zone. Increasing awareness about the interplay between the hydrothermal and magmatic systems might improve volcano monitoring outcomes in the aftermath of large earthquakes.

Monitoring approaches might more specifically address changes at hydrothermal systems and magmatic systems. Ground displacement studies and volcano geodesy, seismicity and tomography, geochemistry and petrology will soon have resolutions high enough to spatially and temporally distinguish the triggered effects at hydrothermal and magmatic systems. Regarding the mechanisms with the magma, current advances in high-temperature experimental facilities now allow for tests to be run at natural conditions. We suggest that this is an important step towards refining our understanding of the processes at stake here. In combination with further and more detailed analysis of ground monitoring and satellite data, the different volcano and hydrothermal effects might become distinguished following different types of earthquakes. Finally, as our records of earthquakes and heightened volcano unrest expands, it remains necessary to regularly update statistical and modelling analyses.

## Data Availability

The authors declare that the data supporting the findings of this study are available within the paper.
